# Books and Authors

**Published:** 1905-10

**Authors:** 


### BOOKS AND AUTHORS.

**Tumors of the Cerebellum.** A Symposium by **Charles K. Mills,** M.D., and others. New York: A. R. Elliott Publishing Co. 1905.

This group of essays comprises: (1) The diagnosis of tumors of the cerebellum, especially with reference to their surgical removal, by Charles K. Mills. (2) Remarks upon the surgical aspects of tumors of the cerebellum, by Charles H.

Frazier. (3) Cases illustrating the papers of Dr. Mills and Dr. Frazier. (4) The ocular symptoms of cerebellar tumors, by George E. de Schweinitz. (5) The pathology of cerebellar tumors, by T. H. Weisenburg. (6) The functions of the cerebellum, by Edward Lodholz. These form, considered together, the most important contribution yet made to the subject of cerebellar tumors.

It is surprising what advances have been made of late in brain surgery. But, it must be observed, that great advances have been made likewise in localising not only functions but new growths of the brain. Localisation of function was, indeed, the first step toward the localising of lesions. With a marvelous precision the lesion, especially if it be a neoplasm or an abscess, is placed, making the responsibility of the surgeon less by that much. If the neurologist be experienced and has located the trouble, the surgeon at once operates in the region pointed out by the former. These two may then work hand in hand, so to speak, in this class of disease.

But there is another specialist who may aid the neurologist in establishing the diagnosis and his work precedes that of the surgeon. We refer to the ophthalmologist who, perhaps, may be consulted first. This will be the case whenever the manifestations of disease begin in the visual apparatus. This brochure is the conjoint work of the neurologist, ophthalmologist, and the surgeon; to which are added the pathologist and physiologist,—a combination hard to defeat.

---

**A Manual of Midwifery.** For Students and Practitioners. By **Henry Jellett,** B.A., M.D., F.R.C.P.L., Gynecologist and Obstetric Physician to Dr. Steevens's Hospital, Dublin. Small octavo, pp. xxv.-1158. 467 illustrations with 9 plates. New York: William Wood & Co. 1905. (Price: muslin, $5.50; sheep, $6.25 net prices.)

Dr. Jellett is not unknown as an author having already written at least two works that have passed two or more editions. His "Short Practice of Midwifery" was well received having already seen its fourth edition, and its associate, the "Short Practice of Gynecology," was issued in its second edition more than a year ago. The author's experience as a teacher and as an examiner justifies the expectation that this one will stand the test that must be applied to every such work—namely, that of practical clinical use.

Some of the views set forth by Jellett fall short of the methods insisted upon by obstetricians in this country. For example, in his directions for hand disinfection he is satisfied with less stringent measures than most generally are insisted upon by teachers here. However, it is likely that differences on this point will always exist since it may be regarded as doubtful whether any method will render the hands absolutely sterile.

This is by far the largest obstetric handbook we have seen.

It covers almost every phase of the subject but is not bulky. By an ingenious use of thin, though excellent paper, a book of nearly eleven hundred and sixty pages is produced without making a cumbersome volume. It is, at the same time, profusely illustrated with high class engravings, which bring out with clearness the author's ideas regarding many points which he does not like to depend upon the text alone for description.

It is altogether a most satisfactory manual, and one which is likely to obtain favor with teachers and students of obstetrics. It certainly is an addition to the literature of the subject with which it deals and is a safe guide to a practitioner in need of help.

---

**Modern Clinical Medicine.** Edited by **J. C. Wilson,** M.D., Professor of Medicine in the Jefferson Medical College, Philadelphia. Vol. I., Infectious Diseases. Translated from Die Deutsche Klinik, under editorial supervision of Julius L. Salinger, M.D. Octavo, pp. xiv.-925. With 60 illustrations and 2 colored plates. New York and London: D. Appleton & Co. 1905. (Price, $6.00.)

The self-imposed task of editing an American edition of the *Deutsche Klinik* is a labor of the head and heart that challenges admiration. There is no dearth of literature on clinical medicine, or, as the Germans prefer to say, internal medicine, at the present time. Nevertheless, there is always room for good material and we venture to predict a place for this great work.

While it is essentially a German treatise, the American editor and translator each has added entire chapters here and there, upon diseases not dealt with in the original. These chapters in this volume include dengue, yellow fever, vaccinia, vaccination, varicella, Malta fever, and Weil's disease or acute febrile icterus.

The arrangement of this volume is convenient, the most important diseases taking the first places and those less so following in the order of their importance or frequency. It is doubtful if ever before so much space has been allotted to the consideration of infectious diseases, as in this compact volume of nearly nine hundred and fifty pages. Moreover, it is closely printed, without waste space. A moderate use of illustration has been made and a good index has been added.

Professor Wilson, the editor, is an experienced teacher and a clinician of acumen and skill. He had made a fame for himself before this system of medicine was even projected, and it cannot be doubted that "Modern Clinical Medicine" will add further laurels to a reputation already world-wide.

---

**Lea's Series of Medical Epitomes.** Edited by **Victor C. Pedersen,** M.D. Diseases of the Eye and Ear. A Manual for Students and Physicians. By Arthur N. Alling, M.D., Clinical Professor of Ophthalmology in Yale University, and Ovidus Arthur Griffin, B. S., M.D. Duodecimo, 263 pages, with 83 illustrations. Philadelphia and New York: Lea Brothers & Co. ($1.00 net.)

The cardinal facts and these only are presented in this excellent epitome. It is just such a book as a beginner should use. It is a mistake to undertake too much in either ophthalmology or otology in taking up the subjects at first, but this book teaches just enough to sharpen the appetite for more,—a fact that will be appreciated by the thoughtful student. Much of the technic in both branches is beyond criticism, and the entire volume contains enough to serve the purpose of the authors. It is, indeed, a résumé that will equip even a junior general practitioner adequately for practical work.

---

**The Development of the Human Body.** A Manual of Human Embryology. By **J. Playfair McMurrich,** A.M., Ph.D., Professor of Anatomy in the University of Michigan. Second edition, revised and enlarged. Duodecimo, pp. 539. With 272 illustrations. Philadelphia: P. Blakiston's Son & Co. 1904. (Price, $3.00.)

The fascinating study of embryology receives an impetus from such a work as this. It is but little more than two years since we noticed the first edition in these columns, and its early exhaustion indicates the interest taken in the subject. Every teacher of anatomy should not only be familiar with embryology himself, but he should teach it, *in limine,* to his pupils. It should be made the foundation of anatomical study.

Beginning with the spermatozoön, the first part of McMurrich's book, which deals with general development, carries the subject up to the development of the fetal membranes,—160 pages; then comes organogeny which, beginning with the skin, takes the student of the subject through the development of the several tissues and systems to post-natal development, to which the final chapter is devoted.

McMurrich is not dull in his presentation of the subject, nor is he verbose. He writes clearly and directly, in excellent form and tells all that is known on each subdivision of human embryology. The illustrations are many and explain the text here and there with precision. We cannot speak otherwise than in high praise of the volume and regard it as a necessity for every teacher and student of anatomy.

---

**Medical Philology.** Gathered by **L. M. Griffiths,** M.R.C.S., Eng. Part I. A-EI. Bristol: J. W. Arrowsmith. 1905.

This little book contains a collection of most interesting words and phrases of medical significance from the Promptorium and Catholicon issued by the Camden Society upon which Mr. Griffiths has made interesting comments. It is a book that will serve to beguile an occasional idle half-hour, and at the same time impart useful information as to the origin of many words rarely used or obsolete; or, again, that have become vulgarised or corrupted. These little essays, for that is what they really are, first appeared in the *Bristol Medico-Chirurgical Journal* and have now been collected in this attractive form, which is an excellent contribution to medical philology. This volume pursues the study only from "A" to "El," but we hope Mr. Griffiths will continue his work to the end of the alphabet.

---

**A Handbook of Nursing.** For Hospital and General Use. Published under the Direction of the Connecticut Training School for Nurses. Duodecimo, pp. 319. Philadelphia and London: J. B. Lippincott Co. 1905.

The occupation of the nurse is one of the most important in which woman can engage, and she must be well instructed at the outset of her career in the ethics of nursing as well as in the duties of her calling. In this handbook the essentials of each,—ethics and duties,—are taught, and with a directness and perspicuity that is as charming as it is instructive.

A properly equipped nurse will be found supplied with several works of this kind, because one is superior in one direction and another in a different way. This one has been in evidence since 1878, having been revised to meet the progression that is taking place in this as in all other professional fields. We commend it to every nurse, and we think it useful also for physicians.

---

**American Edition of Nothnagel's Practice.** Malaria, Influenza, and Dengue. By **Dr. J. Mannaberg,** Vienna, and **Dr. O. Leichtenstern,** Cologne. Edited with additions, by Ronald Ross, F.R.C.S., F.R.S., Professor of Tropical Medicine, University of Liverpool; J. W. W. Stephens, M.D., D.P.H., Walter Myers Lecturer in Tropical Medicine, University of Liverpool; and Albert S. Grünbaum, F.R.C.P., Professor of Experimental Medicine, University of Liverpool. Octavo, 769 pages. Illustrated, including eight full-page plates. Philadelphia and London: W. B. Saunders & Co. 1905. (Cloth, $5.00 net; half morocco, $6.00 net.)

The ever growing interest in the topics discussed in this treatise, especially malaria and influenza, makes this volume particularly acceptable to the American physician. The studies of the relation of malaria to the mosquito are highly interesting and will serve to stimulate into greater activity the somewhat feeble attempts that are making in the destruction of the mosquito in malarial regions.

If the section on influenza is less interesting than the malarial division, it is because, perhaps, of the difference in the nature of the malady itself, and not because it is less ably or less adequately dealt with. In no other place have we seen such an exhaustive and instructive handling of influenza as in this book. The history of the pandemic of 1889-90 which encircled the globe is absorbingly interesting, as is also the general history of the disease. The rapidly contagious character of the infection is astonishing, whole villages being stricken within a few days from the first appearance of the disease.

Dengue, which has some characteristics in common with influenza, is a less common disease. Though highly contagious it is comparatively harmless, the mortality being very slight. Only rarely is it complicated or do sequelæ develop.

This volume is one of the most interesting of this great series and will be welcomed by every physician who desires to keep himself well informed.

---

**Clinical Treatise on the Pathology and Therapy of Disorders of Metabolism and Nutrition.** By **Prof. Dr. Carl von Noorden,** Physician-in-chief to the City Hospital, Frankfurt a. M. Translated under the direction of Boardman Reed, M.D., Professor of Diseases of the Gastrointestinal Tract, Hygiene and Climatology, Department of Medicine, Temple College, Philadelphia. Part VI., Drink Restriction, particularly in Obesity. New York: E. B. Treat & Co. 1905. (Price, 75 cents.)

The title of this book will strike most persons as rather odd, but after all it describes the text with laconic precision. The subject of fluid intake with reference to health, like most other questions, admits of two distinct and opposite viewpoints. No doubt many,—a great many,—individuals drink too little water for their own welfare. This is so marked in many instances that physicians find it necessary to lay the foundation of cure by increasing the quantity of water to be drunk daily by patients suffering from certain maladies.

It is quite true, on the other hand, that many conditions are met in which it becomes equally necessary to restrict the fluid intake with absolute insistence. It is in these disorders that von Noorden's essay applies. Great judgment, however, is required in carrying out drink restrictions; for, whereas, in one and the same person, there may exist a reason for limiting the fluid intake, there also may be equally cogent reasons for not doing so. Hence these methods must be applied with discretion after careful observation.

Von Noorden, nevertheless, has presented a subject in this little monograph that deserves the attention of every clinician and we commend it for its scientific delineation of an unusual topic.

---

**Maternitas. Care of Prospective Mother and Her Child.** By **Charles E. Paddock,** M.D. Professor of Obstetrics, Chicago Post-Graduate Medical School. Duodecimo, pp. 189. Chicago: Cloyd J. Head & Co. 1905. (Price, $1.25.)

Every prospective mother may read such a book as this with great propriety; indeed, it may be asserted that, in view of our present civilisation, it is almost a necessity for her to do so.

Though this one is written by a teacher of obstetrics it is in no sense a scientific treatise. It is, on the other hand, a plain presentation of the simpler facts pertaining to the hygiene of pregnancy, puerperal convalesence, and the care of the baby, that every married woman should be familiar with. By and large it is one of the best books to place in the hands of mothers as well as those about to become mothers that has yet been offered for that purpose. Physicians will do well to recommend it to this end.

---

**Transactions of the American Dermatological Association.** Twenty-eighth annual meeting held at Niagara Falls, N.Y., June 2 and 3, 1904. Charles J. White, M.D., Secretary. New York: The Grafton Press.

This volume records the work of the Association at Niagara Falls in June, 1904, under the presidency of Dr. Joseph Zeisler, of Chicago. The first paper is by Grover W. Wende, of Buffalo, and is a pathological analysis of the famous rhinophyma case first published and illustrated in this JOURNAL, August, 1895. An excellent paper by William Thomas Corlett, of Cleveland, relates to post-vaccinal eruptions. Indeed, the entire volume of ingly interesting, as is also the general history of the malady. of the skin, presented in a most instructive manner.

---

**Conservative Gynecology and Electro-Therapeutics.** A Practical Treatise on Diseases of Women and their Treatment by Electricity. By **G. Betton Massey,** M.D., Attending Surgeon to the American Gynecologic Hospital, Philadelphia. Fourth edition revised. Illustrated. Pages xvi.-468. Octavo. Philadelphia: F. A. Davis Company. 1905. (Cloth, $4.00 net.)

If any physician desires to substitute electricity for the ordinary gynecologic methods of treatment he will find this book full of hints on the subject. It has been revised and to a considerable extent rewritten to meet supposed electrotherapeutic progress. The illustrations are all that could be desired and many of them are new to this edition. The clinical plates are excellent reproductions of patient, physician and nurse, engaged in actual work at the bedside. If this book is read by an experienced gynecologist it will serve to entertain him and add to his library collection, but we submit that it is out of place in the hands of a student. It may only be of service to the advanced practitioner of medicine.

**Report of the Commissioner of Education for the Year 1903. William T. Harris,** Commissioner. Volumes I. and II. Washington: Government Printing Office. 1905.

The present commissioner of education of the United States, William T. Harris, Ph.D., LL.D., came into office September 12, 1889, and is the fourth incumbent since the department was created in 1867. Previous to that date it was called a bureau. Dr. Harris has been identified with education interests for more than thirty years and is an exponent of modern methods as well as everything that makes for improvement and progress in educational affairs.

The two volumes constituting this report covering the calendar year 1903, contain 2,511 pages, embracing material pertaining to almost every phase of education. Naturally that relating to medical schools and medical instruction possesses the greatest interest for physicians, although there is much other material in the report that would well repay examination. In the year 1903 there were 27,062 medical students in the United States,—241 more than 1902. The increase in the "regular" schools was 400; while the decrease in the homeopathic students was 89 and in the eclectics, 70. In the same year there were 5,047 graduates in regular medicine, 419 in homeopathic medicine and 145 eclectics. The total number of homeopathic students was 1,462, and of eclectics, 753. The whole number of medical schools was 146, of which 118 were regular, 19 were homeopathic, and 9 were eclectic.

The report teems with most interesting text and statistical tables that will prove of value to every one who wishes to study the educational problem.

---

**Saunders's Question Compends.** Essentials of the Practice of Medicine. Prepared especially for students of medicine. By **William R. Williams,** M.D., Tutor in Therapeutics, College of Physicians and Surgeons, New York. Duodecimo, 461 pages. Philadelphia and London: W. B. Saunders & Company. 1905. (Double number. Cloth, $1.75, net.)

It is a little surprising that the subject dealt with in this compend,—the practice of medicine,—should not have been taken up in this form before.

It is, nevertheless, a new volume in the series and, moreover, is a double number, though the price is not quite doubled. The author handles his subject well, and we fail to comprehend how he could get more material of value into the space he has allowed himself. The more important data of the principal diseases have been brought out, symptomatology and treatment have been duly set forth and it is, indeed, a book of "essentials" in its truest sense.

---

**Eighteenth Annual Report of the State Board of Health of the State of New Hampshire,** for the two years ending November 1, 1904. Irving A. Watson, M.D., Secretary. Concord: Rumford Printing Company.

The State of New Hampshire takes an advanced position in public health measures and its publications indicate progress in all directions. Its state laboratory of hygiene under the directorship of the secretary of the state board of health is accomplishing excellent work and is a valuable adjunct to the preventive measures heretofore employed. It helps to detect early tuberculosis, to diagnosticate diphtheria, to prevent food adulteration, and to keep water supplies in potable condition. This report is a credit to the state that has furnished a hospitable domicile for the peace plenipotentiaries who lately have evolved the "Treaty of Portsmouth."

